# Development of a Novel Serum Exosomal MicroRNA Nomogram for the Preoperative Prediction of Lymph Node Metastasis in Esophageal Squamous Cell Carcinoma

**DOI:** 10.3389/fonc.2020.573501

**Published:** 2020-10-06

**Authors:** Tong Liu, Lu-Tao Du, Yun-Shan Wang, Shan-Yu Gao, Juan Li, Pei-Long Li, Zhao-Wei Sun, Helen Binang, Chuan-Xin Wang

**Affiliations:** ^1^Department of Clinical Laboratory, The Second Hospital, Cheeloo College of Medicine, Shandong University, Jinan, China; ^2^Shandong Engineering & Technology Research Center for Tumor Marker Detection, Jinan, China; ^3^Shandong Provincial Clinical Medicine Research Center for Clinical Laboratory, Jinan, China; ^4^Department of Surgery, Affiliated Hospital of Shandong University of Traditional Chinese Medicine, Jinan, China; ^5^Department of Surgery, The Affiliated Hospital of Medical College Qingdao University, Qingdao, China

**Keywords:** lymph node metastasis, exosomal biomarker, microRNA, esophageal squamous cell carcinoma, nomogram

## Abstract

Preoperative prediction of lymph node (LN) metastasis is accepted as a crucial independent risk factor for treatment decision-making for esophageal squamous cell carcinoma (ESCC) patients. Our study aimed to establish a non-invasive nomogram to identify LN metastasis preoperatively in ESCC patients. Construction of the nomogram involved three sequential phases with independent patient cohorts. In the discovery phase (*N* = 20), LN metastasis-associated microRNAs (miRNAs) were selected from next-generation sequencing (NGS) assay of human ESCC serum exosome samples. In the training phase (*N* = 178), a nomogram that incorporated exosomal miRNA model and clinicopathologic was developed by multivariate logistic regression analysis to preoperatively predict LN status. In the validation phase (*n* = 188), we validated the predicted nomogram's calibration, discrimination, and clinical usefulness. Four differently expressed miRNAs (chr 8-23234-3p, chr 1-17695-5p, chr 8-2743-5p, and miR-432-5p) were tested and selected in the serum exosome samples from ESCC patients who have or do not have LN metastasis. Subsequently, an optimized four-exosomal miRNA model was constructed and validated in the clinical samples, which could effectively identify ESCC patients with LN metastasis, and was significantly superior to preoperative computed tomography (CT) report. In addition, a clinical nomogram consisting of the four-exosomal miRNA model and CT report was established in training cohort, which showed high predictive value in both training and validation cohorts [area under the receiver operating characteristic curve (AUC): 0.880 and 0.869, respectively]. The Hosmer–Lemeshow test and decision curve analysis implied the nomogram's clinical applicability. Our novel non-invasive nomogram is a robust prediction tool with promising clinical potential for preoperative LN metastasis prediction of ESCC patients, especially in T1 stage.

## Background

Esophageal cancer (EC) is one of the malignant tumors worldwide, which represent the sixth leading cause of cancer-related mortality globally (1). Among the two main histological subtypes of EC, which include esophageal adenocarcinoma (EAC) and esophageal squamous cell carcinoma (ESCC), ESCC accounts for more than 80% of all ECs (2) and is extremely widespread in East Asia, particularly in China (3, 4).

Because the esophagus is anatomically interspersed to the cardiopulmonary organ, ESCC, which has a lymph node (LN) metastasis, causes significantly worse outcomes than do other types of cancers (5). Consequently, LN metastasis is therefore recognized as being the most significant independent risk factor for ESCC prognosis, with overall survival (OS) rates decreasing from ~70 to ~18% when LN metastasis occurs (6). Moreover, appropriate treatment decision-making such as radiotherapy and chemotherapy for patients, surgery involving radical esophagectomy or less invasive endoscopic tumor resection, and determining the region of lymphadenectomy depends primarily on whether or not the tumor has undergone LN metastasis (7–9). Therefore, accurate detection of LN metastasis plays crucial roles in making treatment strategies and patient prognosis (10).

Current LN metastasis detection methods fail from being a gold standard for various reasons. Image methods, such as computed tomography (CT), is often applied to predict LN status preoperatively; however, they also have been observed to be inaccurate in ~40% cancer patients in view of they cannot detect micro-metastasis, which often result in false diagnosis and subsequent inadequate therapy (11). Although high-risk clinical and histopathologic characteristics, including lymphovascular invasion, high T stage, and poor differentiation, are often known as forecasters of LN metastasis (12), these information can only really be given after operation. Thus, clinicians urgently need novel non-invasive biomarkers that can improve LN metastasis detection for reaching more accurate decisions for optimal treatment and improvement in ESCC patients' prognosis.

Exosomes are microvesicles ranging from 30 to 150 nm in size released into the microenvironment by various cell types, especially in cancer progression (13). Exosomes contain proteins, RNAs [including microRNAs (miRNAs)], and lipids; and their cargo often varies under various pathological conditions, being reflective of the physiological state of the originating host cells, which made exosomes act as one of the crucial projects in precision medicine and liquid biopsy (14, 15). Hence, exosomes are a promising source of non-invasive biomarkers for diagnosis, prognosis, and recurrence monitoring of ESCC (16, 17). MiRNAs are small non-coding RNAs (18–26 nt) that target 3′-untranslated regions (3′-UTRs) of mRNAs, leading to posttranscriptional regulation and mRNA destabilization (18). Moreover, a recent study also suggested that dysregulation of various miRNAs is closely related to tumor formation, progression, and metastasis (19). Compared with the unstable features of mRNA and long non-coding RNA (lncRNA), methylated modification of cell-free DNA, and low-abundant circulating RNA (circRNA), exosomal miRNAs are stable with reasonably advanced examination methods, allowing to be suitable predictive biomarkers for various illnesses, including cancer (20). For instance, miR-192, miR-25-3p, miR-17-5p, and miR-122 are enhanced in different tumor tissues and uniformly released into the medium through exosomes (21). In prostate cancer, a circulating five-miRNA signature was identified as useful for differentiating indolent and aggressive forms (22). Our previous studies revealed that miR-203 and miR-200c could be high predictive agents of colorectal cancer (23, 24). While several experiments have proposed that circulating miRNAs are predictive metastasis biomarkers, relatively few have tried to establish a serum exosomal miRNA-based model to predict LN metastasis preoperatively (25, 26). Besides, our group has recently established serum miRNA models to assess preoperative LN conditions in gastric cancer and colorectal cancer (27, 28). Nonetheless, at present, there is no definite proof to show if a serum exosomal miRNA model could improve the forecasting of LN metastasis in ESCC.

The combined analysis of multiple targets could offer robust efficiency compared to single factors because it integrates the effect of several miRNAs and therefore enables superior the diagnostic, prognostic, and predictive performance in the clinical practices (29, 30). In this study, we performed a systematic and comprehensive profiling and quantitative real-time polymerase chain reaction (qRT-PCR) assay of the serum exosomal miRNAs associated with LN metastasis, and then we developed a novel exosomal miRNA model in clinical cohort. The serum exosomal miRNA-based model was subsequently combined with clinical characteristics for constructing a nomogram to predict LN metastasis before surgery. In addition, we verified the predictive performance and clinical usefulness of the nomogram, followed by comprehensive validation in another independent clinical cohort.

## Materials and Methods

### Study Design

**Figure 1:**
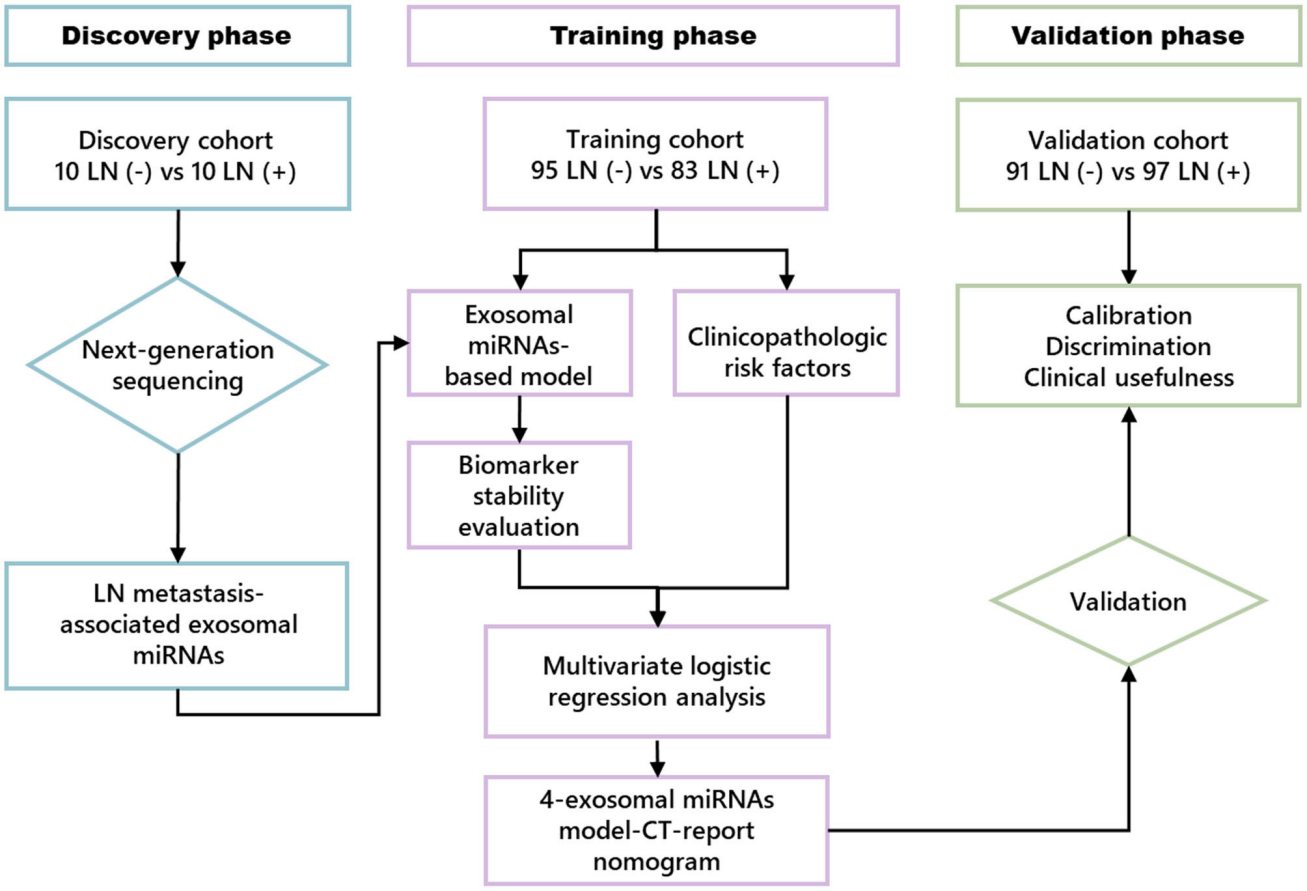
Study flowchart.

Our study was performed in three phases; a flowchart of the study design is illustrated in Figure 1. In the discovery phase, next-generation sequencing (NGS) assay was used in serum exosome samples collected from 10 patients with LN metastasis (LN+) and 10 patients without LN metastasis (LN–) to identify LN metastasis-related exosomal miRNAs.

In the training phase, 17 candidate miRNAs were first tested using qRT-PCR assay in 32 LN– and 32 LN+ ESCC patients' serum exosome samples. Next, miRNAs with Ct values < 35 were further examined in additional samples, including 63 LN– and 51 LN+ patients. This combination cohort (cohort 1) was used in training phase to establish the model for LN status prediction. Receiver operating characteristic (ROC) curve and the area under the ROC curve (AUC) were used to evaluate the discriminative efficiency of exosomal miRNA model for the prediction of LN metastasis. To further explore the predictive value, multivariate logistic regression analysis was employed to assess clinical characteristics, which were substantially correlated with LN metastasis and incorporated with the exosomal miRNA model to establish an LN metastasis prediction nomogram using cohort 1. Subsequently, the performance of the comprehensive nomogram was assessed in cohort 1. In the validation phase, the coefficients of the nomogram from the training set were applied to another independent clinical cohort (cohort 2) consisting of 91 LN– and 97 LN+ patients to validate the predictive performance of this clinical nomogram.

The value of this clinical nomogram was evaluated by three model quality measurements, including calibration, discrimination, and clinical usefulness (31). In this study, we used the Hosmer–Lemeshow test to evaluate the goodness of fit of the clinical nomogram, and the calibration of the clinical nomogram was assessed with the calibration plot. ROC curve analysis was used to indicate the nomogram's discriminative ability. Also, we used decision curve analysis to assess the clinical usefulness of this nomogram in training and validation sets (32).

### Clinical Specimens and Control Subjects

A total of 386 patients who underwent esophagectomy for the treatment of ESCC at the Department of General Surgery of The Second Hospital, Cheeloo College of Medicine, Shandong University, and the Department of General Surgery of Qilu Hospital, Cheeloo College of Medicine, Shandong University, between November 2016 and December 2019 were enrolled in this study. Three forms of esophagectomy were used based on the site of the tumor: the McKeown technique for upper tumors, the Ivor Lewis method for middle tumors, and the Sweet method for lower tumors (12). The method of choice always relies on the discretion of the surgeon. None of the patients had any distant metastasis, underwent any therapies (chemotherapy or radiotherapy) before surgery, or suffered from other malignant disorders at the same time. The preoperative examination, including laboratory tests [SCC and carcinoembryonic antigen (CEA)], endoscopy, and standard CT scan, were performed <2 weeks before surgery. CT-reported LN status was assessed by two radiologists with ≥10 years of experience. The largest regional LN with the short axis diameter > 1 cm was regarded as clinical positive nodal status, and patients without enlarged LNs were defined as clinical negative nodal status (33). Tumor stage was defined according to the classification of American Joint Committee on Cancer (AJCC, 8th edition). All clinical characteristics information, including tumor size, location, differentiation, T stage, LN status, and lymphovascular invasion, were obtained from the postoperative pathology reports by two pathologist. This study was approved by Clinical Research Ethics Committee of The Second Hospital, Cheeloo College of Medicine, Shandong University; and informed consent was received from each participant.

### Serum Sample Preparation, Exosome Purification, and Identification

Serum extracted form blood samples using 3,000 rpm for 15 min at 4°C, followed by centrifugation at 12,000 rpm for 20 min at 4°C (34). Each supernatant was then stored at −80°C until use; 63 μl of ExoQuick™ solution (EXOQ5A-1, SBI System Biosciences, USA) was well mixed with 250 μl of serum and then incubated at room temperature for 30 min. Subsequently, exosomes were extracted by centrifugation 1,600 *g* for 30 min at 4°C. Sediments of exosome were resuspended in 25 μl of saline solution.

The morphology and ultrastructure of exosomes were analyzed using transmission electron microscopy (TEM; JEM-1-11 microscope, Japan). Exosome quantification and size distribution analyses were performed using the ZetaView instrument and software (Particle Metrix Ltd. Germany). Western blot analysis was performed as previously described (35) to determine exosome-specific proteins TSG101 (Abcam, #ab125011) and CD9 (CST, #13403).

### Preparation of Exosomal Small RNA Library and Sequencing

Total RNA from both 10 LN– serum exosome samples and 10 LN+ serum exosome samples were extracted using the Ambion mir Vana miRNA Isolation Kit (Thermo Fisher Scientific, Waltham, USA). The quality of total RNA was examined by Bioanalyzer 2100 (Agilent Technologies, Santa Clara, USA), and the concentration of the total RNA was tested using NanoDrop 2000 (Thermo Fisher Scientific, Lafayette, USA). In the present study, miRNA libraries were established as follows: about 500 ng of total RNA of individual sample was used for constructing cDNA library through the TruSeq Small RNA Preparation Kit (Illumina, San Diego, USA) according to the manufacturer's instruction. Further, the cDNA libraries were sequenced with single-end 50 bp (SE50) by Illumina HiSeq 2500 platform (Lc-bio, Hangzhou, China) as described in previous research (36). The raw and processed data have been deposited into the Gene Expression Omnibus (GEO) database (https://www.ncbi.nlm.nih.gov/geo/) under accession number GSE155360.

### Data Filtering and Identification of Novel Candidate MicroRNAs

System and comprehensive data filtering steps were performed after obtaining the raw reads. The raw miRNAs read were analyzed using the in-house program ACGT101-miR (LC Sciences, Houston, USA) to remove adapter dimers, junk, low complexity, common RNA families [ribosomal RNA (rRNA), transfer RNA (tRNA), small nuclear RNA (snRNA), and small nucleolar RNA (snoRNA)], and repeats according to the manufacturer's instruction.

To identify novel 5p- and 3p-derived miRNAs and known miRNAs, unique sequences with lengths of ~18–26 nucleotides were mapped to specific species precursors in miRBase 22.0 by BLAST search as previously described (37, 38). Briefly, the unique sequence mapping to specific species' mature miRNAs in hairpin arms identified known miRNAs. Then the unique sequence mapping to the other arm of known specific species precursor hairpin opposite to the annotated mature miRNA-containing arm considered be novel 5p- or 3p-derived miRNA candidates. Furthermore, the remaining sequences were mapped to other selected species' precursors (with the exclusion of specific species) in miRBase 22.0 by BLAST search against the specific genomes, and the sequences containing hairpin RNA structures were predicted from the flank 80-nt sequences using RNAfold software (http://rna.tbi.univie.ac.at/cgi-bin/RNAfold.cgi) in order to predict novel candidate miRNAs more accurately (37, 38).

### Prediction of MicroRNAs' Target and Functional Enrichment Analysis

To explore how the four miRNAs could regulate tumor progression, two mRNA target-predicting algorithms (miRanda and TargetScan) were utilized to identify the potential downstream targets of the four miRNAs (39). To examine the underlying functions of selected miRNA and targeted mRNAs, Kyoto Encyclopedia of Genes and Genomes (KEGG) pathway enrichment analyses and Gene Ontology (GO) terms were calculated using the “clusterProfiler” package in R project software (40). KEGG pathway analysis was performed to clarify pathways related to miRNA and targeted mRNAs. GO analysis assessed molecular functions (MFs), biological processes (BPs), and cellular components (CCs).

### RNA Extraction and qRT-PCR

miRNeasy Micro Kit (QIAGEN, Valencia, CA, USA) was used to isolate exosomal total RNA. Exosome samples were processed according to the manufacturer's instruction as described in a previous study (35). The extracted RNA was resuspended with 16 μl of nuclease-free water. Then we used NanoDrop spectrophotometer (Thermo Fisher Scientific, Lafayette, USA) to evaluate the concentration and quality of the RNA. cDNA was synthesized from 200 ng of template RNA using the Mir-X MiRNA First-Stand Synthesis Kit (Takara, Dalian, China) in a 10 μl of reaction volume.

After 5-fold dilution, 2 μl of cDNA was used for qPCR assay that was reacted using the TB Green™ Premix Ex Taq™ (Takara, Dalian, China) on a CFX96 Real-Time Detection System (Bio-Rad Laboratories, USA). MiRNA primers were synthesized by Ribobio (Guangzhou, China), and forward primer sequence has been provided in Supplementary Table 1. Each qRT-PCR was repeated three times. The relative expression of miRNAs was calculated and normalized to miR-16, using the comparative threshold (2^−ΔΔCt^) method.

### Statistical Analysis

All statistical data were analyzed by MedCalc 9.3.9.0, GraphPad Prism (version 8.0, GraphPad Software, La Jolla, CA, USA), SPSS (version 22.0, Chicago, IL, USA), and R software (version 3.4.2). We used the Kolmogorov–Smirnov test to determine the distribution of each group. Continuous variables were shown in median (interquartile range) or mean ± standard deviation (SD). Categorical variables were presented as count or proportion. The Mann–Whitney *U*-test and Student *t*-test were applied for comparisons of exosomal miRNAs among two groups. ROC curves, calibration plot, and decision curve analysis were performed to indicate the LN metastasis predictive value of the nomogram in ESCC. *P* < 0.05 was considered to be statistically significant.

## Results

### Primary Data Collection and Global Screening of Exosomal MicroRNA Expression

**Figure 2:**
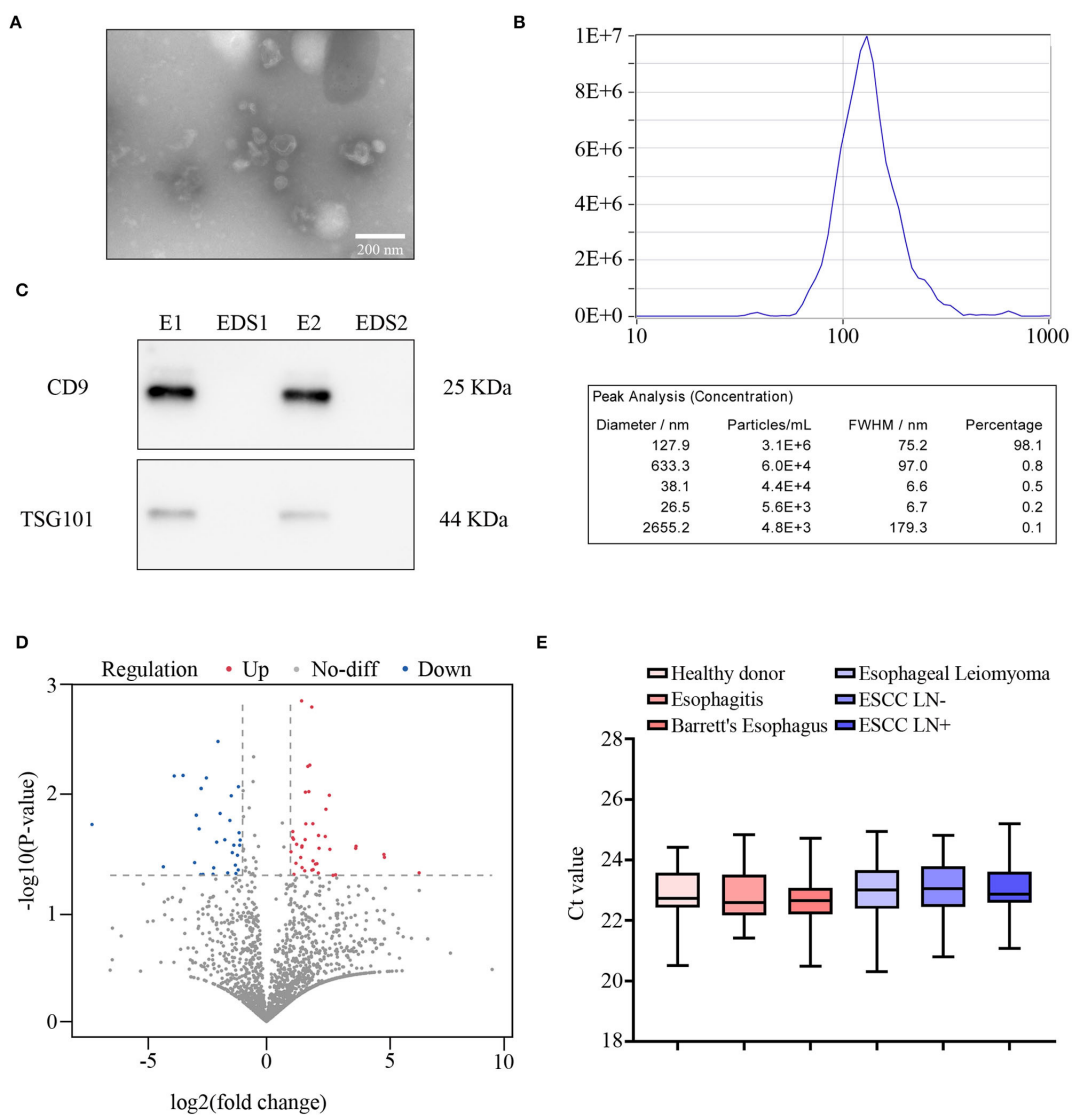
Primary data collection and global screening of microRNA (miRNA) expression to predict lymph node (LN) metastasis. **(A)** The morphology of exosomes was identified using transmission electron microscopy (TEM). Scale bar, 100 nm. **(B)** Size distribution and concentration of exosomes. **(C)** Western blot assay of CD9 and TSG101 in exosome samples (E) and exosome-depleted supernatants (EDSs). **(D)** Volcano plot showing the differentially expressed miRNAs from next-generation sequencing (NGS) analysis. **(E)** Comparison of internal control exosomal miR-16 expression among HD (*n* = 38), reflux esophagitis (*n* = 30), Barrett's esophagus (*n* = 18), esophageal leiomyoma (*n* = 15), LN– (*n* = 30), and LN+ (*n* = 30) detected by qRT-PCR.

Table 1 shows the pathological characteristics and clinical information, which had no significant difference in the distribution between the training and validation sets (all *P* > 0.05). Moreover, preoperative CT report was observed to be significantly correlated with LN metastasis in both cohorts. Exosomes with typical cup-shaped, round morphologies, 30–150 nm in diameters, were detected by TEM (Figure 2A). ZetaView results showed that majority of the serum exosomes were 127.9 nm in diameter (Figure 2B). Increased level of CD9 and TSG101 protein was observed in exosomes as compared with that in exosome-depleted supernatants (EDSs) (Figure 2C). These data showed that those exosomes were isolated from serum samples.

**Table 1:** Clinical characteristics.

**Variable, N. (%)**	**Discovery set**		**Training set**				**Validation set**				
	**LNM (-)**	**LNM (+)**	**Total**	**LNM (-)**	**LNM (+)**	***P***	**Total**	**LNM (-)**	**LNM (+)**	***P***	***P^**#**^***
**Sex**						0.916				0.081	0.837
Male	9 (90)	10 (100)	128 (71.9)	68 (71.6)	60 (72.3)		137 (72.9)	61 (67.0)	76 (78.4)		
Female	1 (10)	(0)	50 (28.1)	27 (28.4)	23 (27.7)		51 (27.1)	30 (33.0)	21 (21.6)		
Age^a^						0.318				0.884	0.333
<62	5 (50)	3 (30)	80 (44.9)	46 (48.4)	34 (41.0)		94 (50.0)	46 (50.5)	48 (49.5)		
≥62	5 (50)	7 (70)	98 (55.1)	49 (51.6)	49 (59.0)		94 (50.0)	45 (49.5)	49 (50.5)		
Tumor location						0.099				0.706	0.736
Cervical-middle thoracic	5 (50)	5 (50)	102 (57.3)	49 (51.6)	53 (63.9)		111 (59.0)	55 (60.4)	56 (57.7)		
Low thoracic	5 (50)	5 (50)	76 (42.7)	46 (48.4)	30 (36.1)		77 (41.0)	36 (39.6)	41 (42.3)		
Differentiation						<0.001				0.070	0.409
Well-moderate	8 (80)	6 (60)	107 (60.1)	70 (73.7)	37 (44.6)		105 (55.8)	57 (62.6)	48 (49.5)		
Poor	2 (20)	4 (40)	71 (39.9)	25 (26.3)	46 (55.4)		83 (44.2)	34 (37.4)	49 (50.5)		
T stage						0.004				0.006	0.700
T1-T2	5 (50)	2 (20)	85 (47.8)	55 (57.9)	30 (36.1)		86 (45.7)	51 (56.0)	35 (36.1)		
T3-T4	5 (50)	8 (80)	93 (52.2)	40 (42.1)	53 (63.9)		102 (54.3)	40 (44.0)	62 (63.9)		
Tumor size^b^						<0.001				<0.001	0.663
<4.0	6 (60)	4 (40)	94 (52.8)	64 (67.4)	30 (36.1)		95 (50.5)	58 (63.7)	37 (38.1)		
≥4.0	4 (40)	6 (60)	84 (47.2)	31 (32.6)	53 (63.9)		93 (49.5)	33 (36.3)	60 (61.9)		
SCC level						0.844				0.530	0.404
Normal	7 (70)	6 (60)	108 (60.7)	57 (60.0)	51 (61.4)		122 (64.9)	57 (62.6)	65 (67.0)		
Abnormal	3 (30)	4 (40)	70 (39.3)	38 (40.0)	32 (38.6)		66 (35.1)	34 (37.4)	32 (33.0)		
CEA level						0.401				0.533	0.477
Normal	8 (80)	7 (70)	113 (63.5)	63 (66.3)	50 (60.2)		126 (67.0)	63 (69.2)	63 (64.9)		
Abnormal	2 (20)	3 (30)	65 (36.5)	32 (33.7)	33 (39.8)		62 (33.0)	28 (30.8)	34 (35.1)		
CT reported LN status						<0.001				<0.001	0.409
Negative	6 (60)	5 (50)	107 (60.1)	70 (73.7)	37 (44.6)		105 (55.8)	67 (73.6)	38 (39.2)		
Positive	4 (40)	5 (50)	71 (39.9)	25 (26.3)	46 (55.4)		83 (44.2)	24 (26.4)	59 (60.8)		
Lymphovascular invasion						0.018				0.002	0.747
Negative	5 (50)	4 (40)	92 (51.7)	57 (60.0)	35 (42.2)		94 (50.0)	56 (61.5)	38 (39.2)		
Positive	5 (50)	6 (60)	86 (48.3)	38 (40.0)	48 (57.8)		94 (50.0)	35 (38.5)	59 (60.8)		

P*: The difference between the Training cohort and Validation cohort.

a *The average age was 62*.

b *Tumor size measured in longest diameter (cm), and the mean was 4.0 cm*.

To develop an exosomal miRNA-based model for prediction of LN metastasis, we first performed genome-wide miRNA NGS-based miRNA expression profiling for 20 serum exosomes of ESCC patients (10 LN– and 10 LN+ cases). As shown in Figure 2D, of the total 6,959 annotated miRNAs, 91 were markedly differentially expressed (*P* < 0.05, absolute log2 fold change > 1, Wilcoxon signed-rank test). Further, to obtain the exosomal miRNA model for the clinical application, we excluded the low-expression-level miRNAs (average expression level < 50), which led to the selection of 17 miRNAs (Supplementary Figure 1A).

Ours and other previous studies suggested that miR-16 could act as an internal control for normalizing exosomal miRNA expression *in vitro* (35, 41). Therefore, we hypothesis that miR-16 could also act as the internal control in serum exosomes. In the present study, melting curve analysis indicated that miR-16 generated a unique peak; and no detectable Ct value was observed in negative controls, which verified the lack of contamination and non-specific amplification (data not shown). Furthermore, no significant differences were found in the expression of exosomal miR-16 among the six different groups [LN–, LN+, reflux esophagitis, Barrett's esophagus, esophageal leiomyoma, and the healthy donor (HD) groups] by using qRT-PCR assay (all *P* > 0.05, Figure 2E). Collectively, these findings show that miR-16 could be applied as a suitable internal control for normalizing serum exosomal miRNAs.

### Evaluation and Identification of Candidate Exosomal MicroRNAs

**Figure 3:**
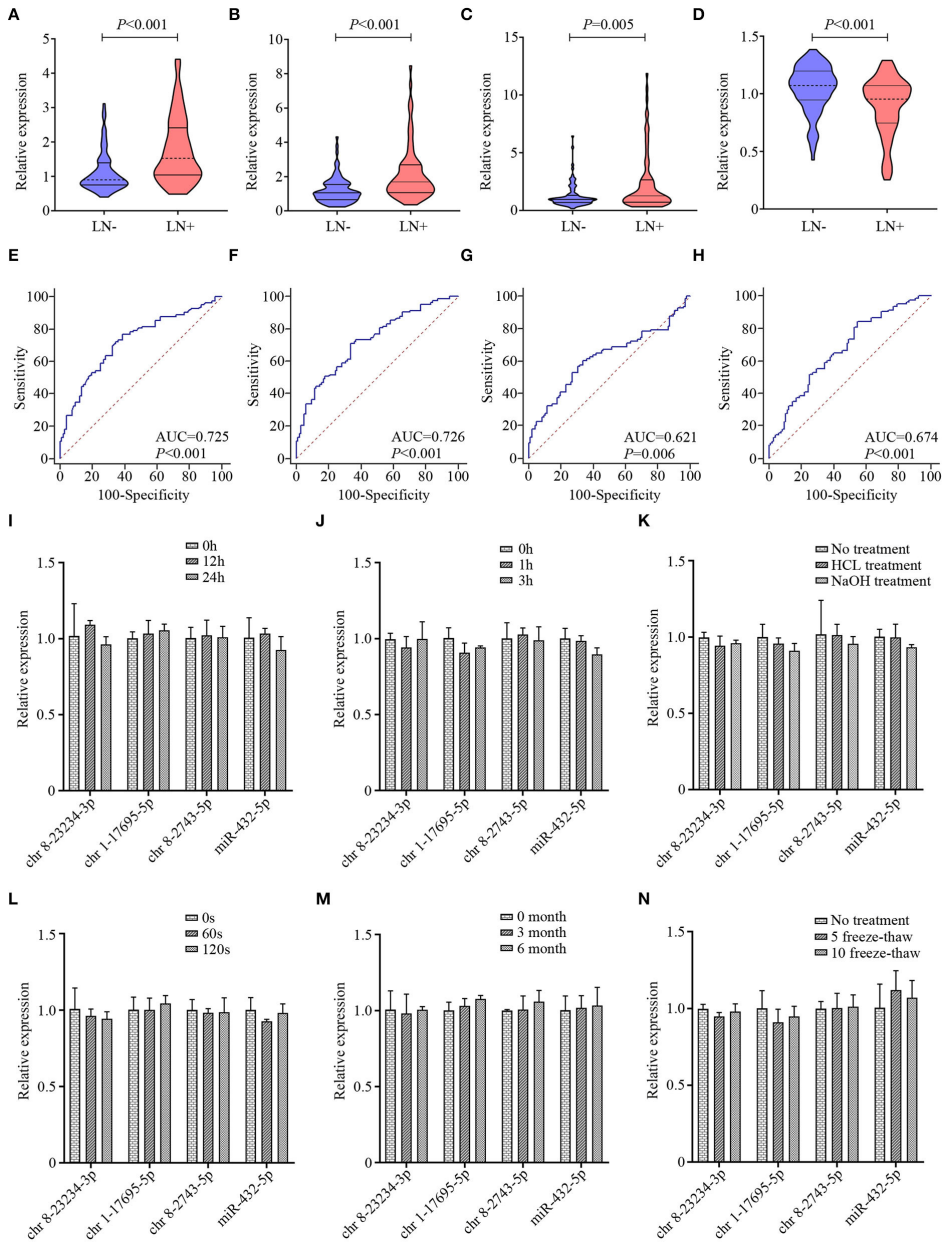
The violin plots and receiver operating characteristic (ROC) curves for exosomal chr 8-23234-3p, chr 1-17695-5p, chr 8-2743-5p, and miR-432-5p in the training cohort. The relative expression level of exosomal chr 8-23234-3p **(A)**, chr 1-17695-5p (**B**), chr 8-2743-5p **(C)**, and miR-432-5p **(D)** were examined by qRT-PCR. ROC curve analysis to predict metastasis using chr 8-23234-3p **(E)**, chr 1-17695-5p **(F)**, chr 8-2743-5p **(G)**, and miR-432-5p **(H)**. Comparison of the four-microRNA (miRNA) expression level before and after harsh treatment including room temperature incubation **(I)**, RNase A treatment **(J)**, acid and base solution treatments **(K)**, vigorous shaking **(L)**, −80°C incubation **(M)**, and multiple freeze–thaw cycles **(N)**.

The 17 candidate miRNAs revealed by NGS were first investigated in clinical serum exosome samples consisting of 32 LN– and 32 LN+ ESCC patients. MiRNAs with the detection rate lower than 75% and/or Ct mean value higher than 35 in both LN– and LN+ groups were excluded from further analysis. Then, further utilizing qRT-PCR, an additional 114 clinical serum exosomal samples from ESCC patients (63 LN–and 51 LN+) were analyzed to validate the above phenomena. Thus, the training cohort comprised 95 LN– and 83 LN+ patients (cohort 1). To avoid any redundancy and overlapping potential predictive miRNAs, we utilized a backward step-wise elimination approach to further eliminate five miRNAs, yielding the final four exosomal miRNA selections. In the training set, chr 8-23234-3p, chr 1-17695-5p, and chr 8-2743-5p were upregulated and miR-432-5p was downregulated in LN+ patients compared with LN– patients (Figures 3A–D). ROC curve analysis revealed that the AUC of chr 8-23234-3p, chr 1-17695-5p, chr 8-2743-5p, and miR-432-5p for LN metastasis prediction ranged from 0.621 to 0.726 (Figures 3E–H). The expression level of these four exosomal miRNAs were further measured using another independent confirmation cohort (cohort 2) with 91 LN– and 97 LN+ patients. The alterations in the miRNAs expression pattern of the validation cohort agreed with those from the training cohort, with AUCs varying from 0.629 to 0.739 (Supplementary Figures 1B–I and Supplementary Table 2). Furthermore, the expression level of four exosomal miRNA in HD serum exosome samples was also investigated. As shown in Supplementary Figures 1J,L, exosomal miRNA chr 8-23234-3p and chr 8-2743-5p were upregulated only when LN metastasis happened. The expression level of chr 1-17695-5p in HD group was lower than in the ESCC group (both LN– and LN+ samples, Supplementary Figure 1K). However, no significant difference was observed in the expression of exosomal miR-432-5p between HD and ESCC group (both LN– and LN+ samples, Supplementary Figure 1M).

The instability of miRNAs in serum remains a significant limitation for clinical application. To investigate whether the exosome membrane could protect exosomal miRNA, exosome sample was subjected to harsh conditions including incubation at room temperature for 0, 12, and 24 h (Figure 3I); incubation with RNase A for 1 and 3 h (Figure 3J); incubation with strong acid–base treatment (Figure 3K); vigorous shaking for 0, 60, and 120 s (Figure 3L); incubation at −80°C for 0, 3, and 6 months (Figure 3M); and multiple freeze–thaw cycles for 0, 5, and 10 (Figure 3N). Total RNA was then isolated, and qRT-PCR was performed to evaluate the stability of these four exosomal miRNAs. Results indicated that these treatments had hardly any effects on the level of exosomal miRNAs. Taken together, these data indicated that exosomal miRNAs were detectable and stable in exosomes, which establishes their sufficient suitability as tumor markers for ESCC LN status prediction.

### Construction and Validation of a Four-Exosomal MicroRNA Model

**Figure 4:**
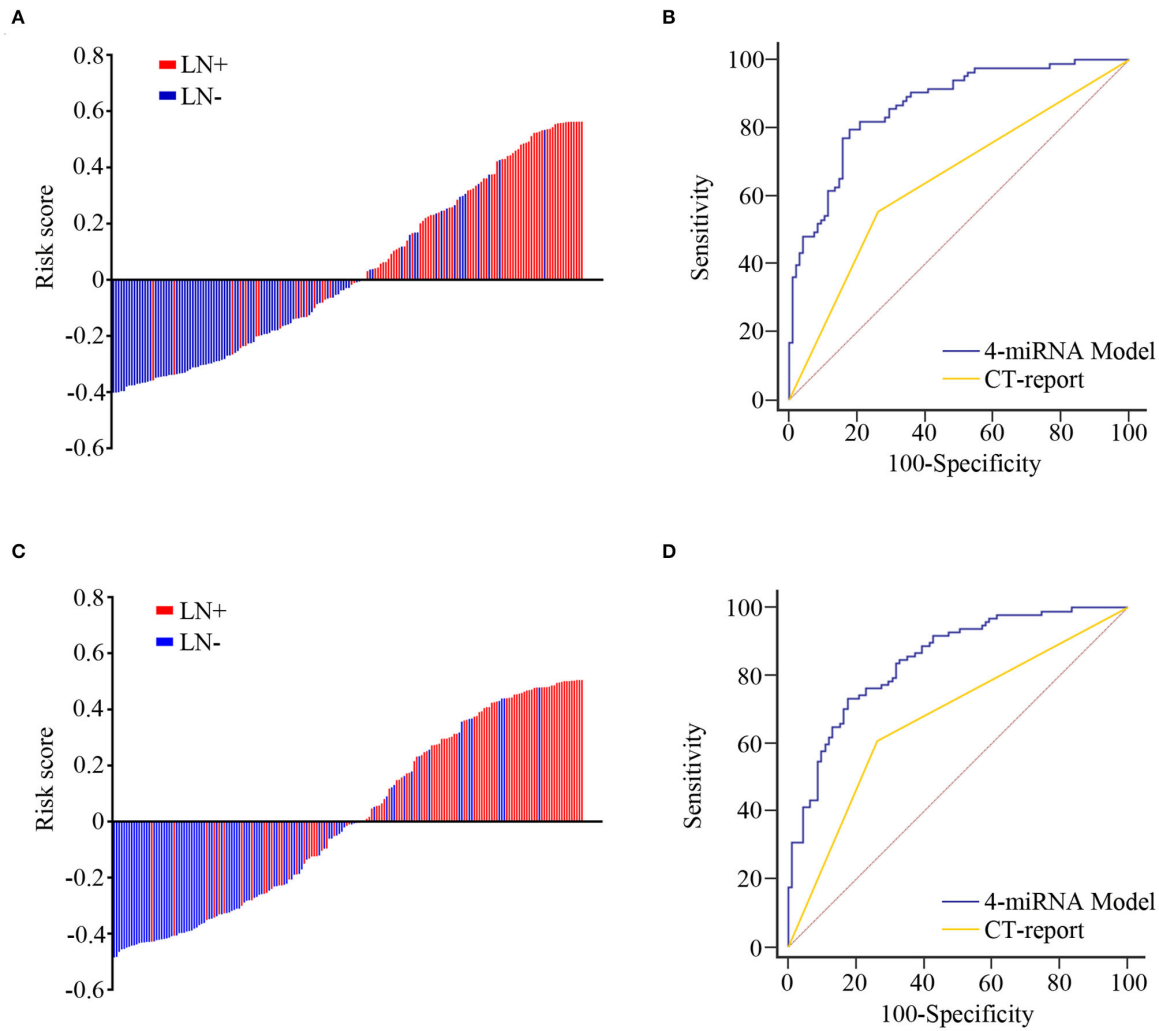
The predictive value of microRNA (miRNA)-based model for lymph node (LN) metastasis in training and validation cohorts. The detection values of the four-exosomal miRNA model in each patient (red line, with LN metastasis; blue line, without LN metastasis) in the training **(A)** and validation **(C)** cohorts. Receiver operating characteristic (ROC) curve analysis for LN metastasis prediction through four-exosomal miRNA model or computed tomography (CT) report in training **(B)** and validation **(D)** cohorts.

Univariate and multivariate logistic regression analyses revealed that each of the four exosomal miRNAs could act as independent predictive factors for LN metastasis in ESCC (Supplementary Figures 2A,B). The predicted probability of LN metastasis from the logit model based on the four exosomal miRNA expression, logit (*P* = LN metastasis) = 1.466 ^*^ chr 8-23234-3p + 0.815 ^*^ chr 1-17695-5p + 0.398 ^*^ chr 8-2743-5p – 3.833 ^*^ miR-432-5p – 0.292. Patients were classified into low- and high-risk categories dependent on the measured risk scores of the Youden index-derived cutoff thresholds. The corresponding four-exosomal miRNA model showed an outstanding efficiency for the identification of LN metastases with an AUC value of 0.865 (95% CI: 0.805–0.911, Figures 4A,B) in the training group. Consequently, we used the same model and coefficients obtained from cohort 1 to separate cohort 2, which once again affirmed the effectiveness of our model in predicting LN metastasis ESCC patients with an AUC value of 0.845 (95% CI: 0.785–0.893, Figures 4C,D). Meanwhile, the AUC for CT-reported LN metastasis was only 0.646 (95% CI: 0.570–0.716) in cohort 1 and 0.672 (95% CI: 0.600–0.739) in cohort 2. Therefore, our four-exosomal miRNA risk model provides better detective potential than conventional CT report in both cohorts (*P* < 0.01).

### Development and Validation of a Clinical Prediction Nomogram

**Figure 5:**
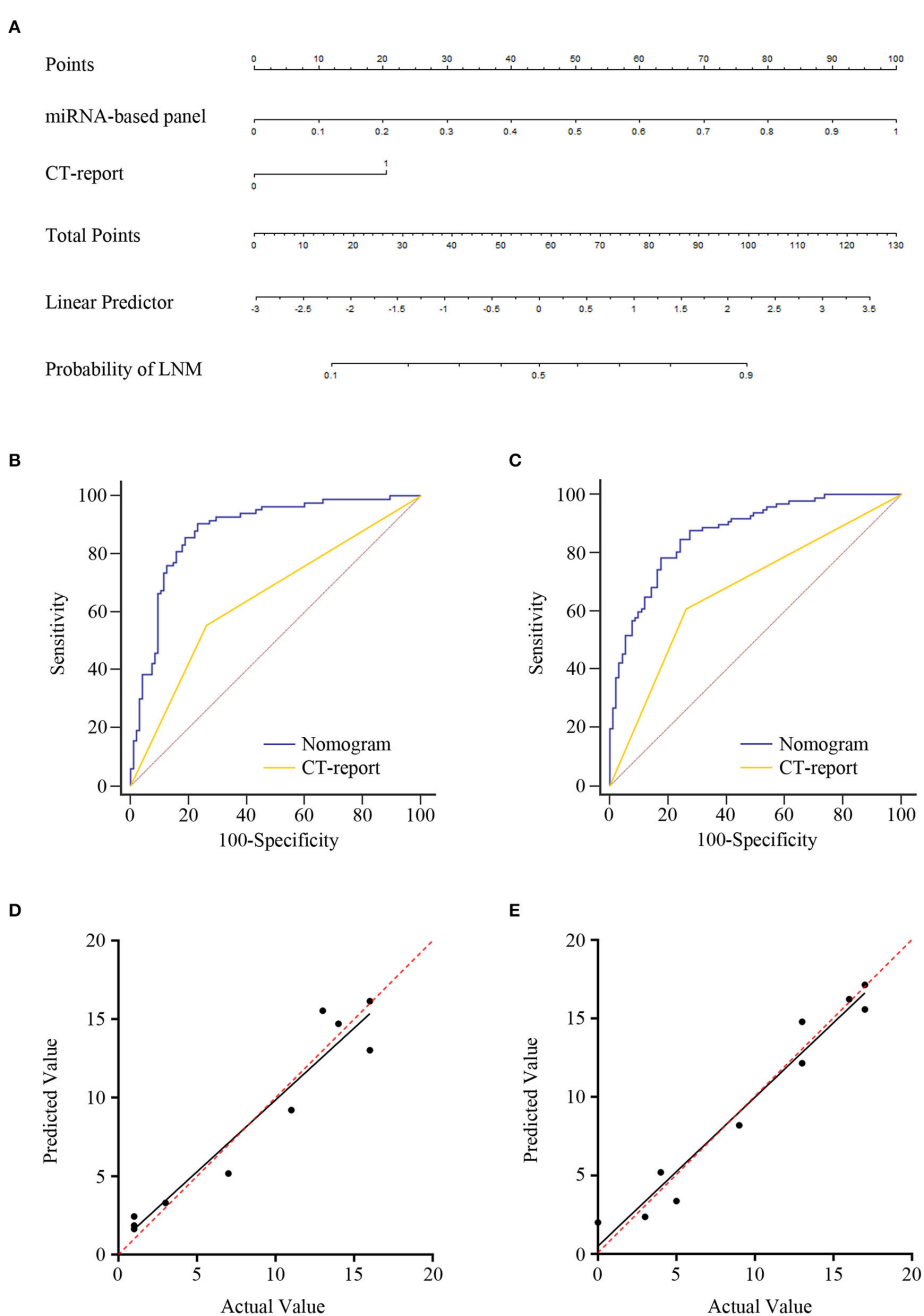
Development of lymph node (LN) metastasis preoperative prediction nomogram and its clinical value. **(A)** Combination four-exosomal microRNA (miRNA) model with computed tomography (CT) report for constructing LN metastasis prediction nomogram of ESCC patients in training cohort (CT report: 0 represents negative and 1 represents positive). Receiver operating characteristic (ROC) curve analysis of the nomogram in the training [**(B)**, AUC = 0.880, 95% CI: 0.822–0.923] and validation [**(C)**, AUC = 0.869, 95% CI: 0.812–0.913] cohorts. Calibration plots of the nomogram in the training (**D**) and validation (**E**) cohorts. The *x*-axis represents actual probability, and the *y*-axis represents nomogram-predicted probability of LN metastasis. The red line with 45° implies an ideal status, and the black line implies the calibration value of the nomogram. The black line that lies close to the red line implies a perfect calibration.

**Figure 6:**
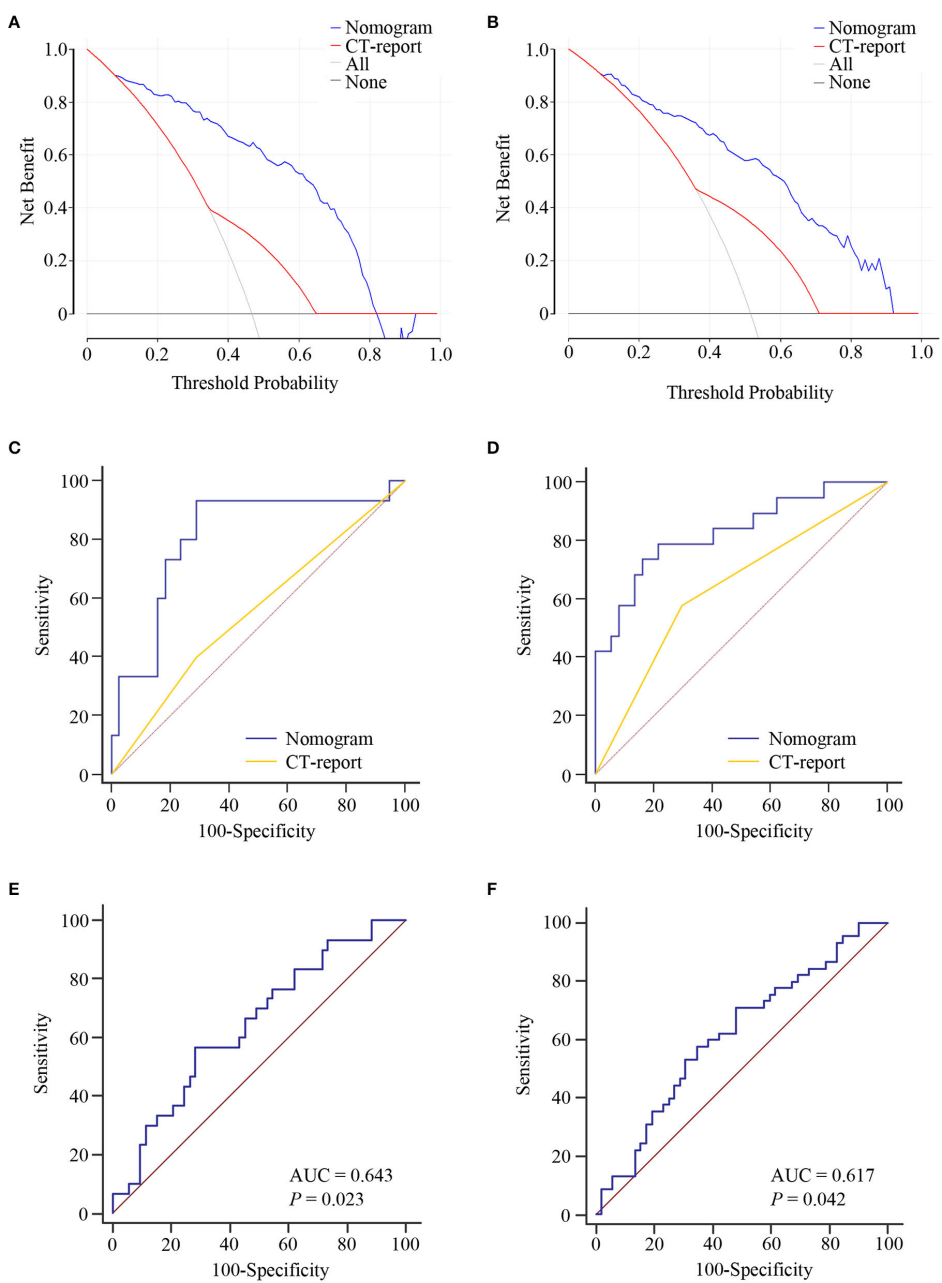
The clinical value of the lymph node (LN) metastasis preoperative prediction nomogram. Decision curve analysis for the nomogram and computed tomography (CT) report in the training **(A)** and validation **(B)** cohorts. Net benefit is plotted on the *y*-axis; threshold probabilities is plotted on the *x*-axis. The blue line represents nomogram; the red line represents CT report; the gray solid line (treat all) represents the assumption that all patients had LN metastasis; the black solid line (treat none) represents no patients had LN metastasis. Receiver operating characteristic (ROC) curve analysis of nomogram for the prediction of LN metastasis in T1 stage of esophageal squamous cell carcinoma (ESCC) patients in the training **(C)** and validation **(D)** cohorts. ROC curve analysis of nomogram for the discrimination of N stage in ESCC patients in the training **(E)** and validation **(F)** cohorts.

Logistic regression assay indicated that our four-exosomal miRNA risk model and the CT report were independent risk factors for LN metastasis (Supplementary Table 3). Based on the above results, we established a clinical nomogram to estimate the risk of LN metastasis (Figure 5A). The AUC of our novel nomogram was 0.880 (95% CI: 0.822–0.923, Figure 5B) for the training cohort. The calibration plot revealed a strong correlation between the actual and predicted values of the training cohort (Figure 5D). The findings of the Hosmer–Lemeshow goodness-of-fit test also was not significant (*P* = 0.321). In compliance with the training cohort, the AUC of the validation cohort was 0.869 (95% CI: 0.812–0.913, Figure 5C). Good calibration of the nomogram was also noted in the testing samples (Hosmer–Lemeshow goodness-of-fit test, *P* = 0.574, Figure 5E). Decision curve analysis indicated that a threshold of 20–80% to guide examination was superior compared with the “treat-all,” “treat-none,” or CT-report scheme (Figures 6A,B).

Furthermore, our nomogram risk stratification was also performed well in predicting LN metastasis in T1 ESCC patients; at the same time, the CT-reported LN status criteria did not work. Compared with CT-reported LN status criteria, our nomogram could successfully identify true, high-risk, T1 stage ESCC patients with excellent accuracy in training AUC = 0.811 (95% CI: 0.679–0.905, Figure 6C) and validation AUC = 0.831 (95% CI: 0.707–0.918, Figure 6D) cohorts. Besides, we noted that our nomogram risk score was correlated with the level of LN metastasis (N1 vs. N2–3 stage). ROC curve was constructed for the evaluation of the nomogram in N stage prediction. The AUC value of 0.643 was observed in the training cohort and 0.617 in the validation cohort (Figures 6E,F). These data highlighted the LN metastasis prediction potential of our nomogram in clinical practices of ESCC.

### Pathway Enrichment Analysis of Predictive Exosomal MicroRNAs

To obtain further insight into the functional mechanism of the four exosomal miRNAs in ESCC, we performed GO categories and KEGG pathway enrichment analysis on the target mRNAs of these miRNAs. GO enrichment analysis of the source gene consisted of BPs, CCs, and MFs (Supplementary Figure 2C). Three significantly enriched GO terms in BPs identified were “signal transduction,” “regulation of transcription,” and “multicellular organism development.” When classified according to CC, the three most significantly enriched GO terms were “membrane,” “cytoplasm,” and “nucleus.” Based on MFs, three significantly enriched GO terms were “protein binding,” “mental ion binding,” and “transferase activity.” The data of KEGG analysis revealed that most target genes participated in cancer pathway, Ras signaling pathway, Rap1 signaling pathway, PI3K-Akt signaling pathway, and MAPK signaling pathway (Supplementary Figure 3). These results suggested that these four exosomal miRNAs investigated in our study may not only identify as premising predictive biomarkers but also serve as potential treatment targets for ESCC.

## Discussion

LN metastasis is a crucial risk factor of prognosis and recurrence in ESCC patients; therefore, identification of LN metastasis is essential for deciding the therapeutic strategies as well as the scope of surgical procedure. However, LN metastasis was frequently misidentified because of the limitations of current predictive methodologies in ~20–40% of ESCC cases (11, 42, 43). Thus, non-invasive molecular biomarkers to accurately and preoperatively predict LN metastasis is direly needed for making the proper treatment procedure of ESCC. In this study, we constructed a novel serum four-exosomal miRNA model for the identification of LN metastasis cases in ESCC patients with high accuracy. Furthermore, a clinical nomogram combining the four-exosomal miRNA model and CT report was developed to identify LN metastasis in ESCC patients, which performed higher predictive efficiency in two independent cohorts, and particularly displayed satisfactory LN metastasis predictive accuracy in T1 stage of ESCC.

Blood extraction is more convenient than esophagography and endoscopic ultrasonography tests because it carries less risk of injury with no esophageal discomfort. Blood-based analyses can be merged into routine blood tests, which could remarkedly reduce examine time for clinical practices. The study of exosomal miRNAs as circulating biomarker is evolving rapidly, and tumor research in this area provides great potential in clinical application. Furthermore, our studies indicated that exosomes are stable, are resistant to physical and chemical treatments, and can be stored for extended periods without significant degradation of encapsulated miRNAs. This property of exosomes increases its potential applicability in the laboratory/clinical interface. Besides, tumor cell-derived exosomal miRNAs are reflective of their originating host cells and therefore may offer tumor-related profiles that are more specific than the miRNA profile of whole blood or even serum (44). Therefore, exosomal miRNAs are promising non-invasive tumor markers with great potential to be applied in individualized treatment. Although previous studies have made some advances, lots of researches have only focused on a few pre-identified and individual miRNAs, leaving a large number of miRNAs being neglected. In the discovery phase of this study, differently expressed miRNAs were firstly selected in serum exosomes from ESCC patients with or without LN metastasis using an NGS-based miRNA expression profiling assay, which enabled us to have better chance to identify potential predictive biomarkers. From these data, candidate miRNAs revealed by NGS assay were further evaluated by two independent clinical cohort validations. As far as we know, this is the first comprehensive and systematic research for identifying LN metastasis biomarkers based on serum exosomal miRNA expression assay in ESCC patients before surgery.

In the present study, we hypothesized that an integrated nomogram combining exosomal miRNA model and clinical characteristics could increase the accuracy of LN status prediction. Therefore, we established a risk score assessment formula of the four-exosomal miRNA model that enables clinicians to obtain superior predictive efficacy compared with conventional CT report. Subsequently, our newly developed exosomal four-miRNA model together with the determination of CT report can enhance the detection accuracy of LN metastasis in ESCC. Through this study, we suggested that the potential of a combined clinical nomogram for preoperative prediction of LN metastasis among ESCC patients is achievable and could be applied to clinical practice in the future.

From a clinical standpoint, LN metastasis serves a critical role in optimizing individually tailored therapy, especially in ESCC patients of the T1 stage. In the absence of LN metastasis, Tis, T1a, and T1b (optional) ESCC patients can be effectively managed with endoscopic mucosal or submucosal resection. However, if the LN metastasis happened, radical operation with lymphadenectomy could improve the patient's prognosis. Additionally, more precise information of LN status could be beneficial for preoperative chemo(radio)therapy decision-making, as suggested by the National Comprehensive Cancer Network (NCCN) guidelines (version 2019) (45). More important, as the total number of surgical resected LNs increases, the number of confirmed metastatic LNs also increases. Therefore, insufficient resected LNs might result in underestimation of the disease severity and subsequent tumor progression (46). Meanwhile, adequate resection of LN by the radical operation is a technical challenge in clinical practices. However, radical LN dissection also increases morbidity and mortality after surgery, especially for cardiopulmonary diseases and elderly patients (2, 47). Hence, the availability of preoperative high-accuracy biomarkers for LN metastasis prediction will optimize individually tailored surgical intervention and ultimately reduce mortality and morbidity in patients with ESCC by preventing excessive lymphadenectomy. In this research, our clinical nomogram showed comparable AUCs when tested in the T1 stage, and also in patients who had not undergone neoadjuvant chemotherapy or chemoradiation therapy, which highlight the consistency of our model. Furthermore, ESCC patients with a high risk score of nomogram had higher numbers of metastasis LNs than those with a low risk score, which means that our clinical nomogram has the potential to provide clinicians with a relatively accurate N stage prediction information. These results suggest that using our clinical nomogram to direct decision-making on esophagectomy and lymphadenectomy would boost patients' health outcomes.

The most important part for adopting the nomogram into clinical use is to certify whether nomogram-assisted treatment decisions could improve patient outcomes. Nonetheless, adequate calibration and discrimination are not enough for a predictive model to be clinically beneficial (48), and most researchers have not performed further analysis of the clinical utility of the model. Also, it may not be appropriate to artificially select a risk threshold because the doctor and patient may have different risk thresholds. Therefore, our predictive nomogram's clinical value was estimated with decision curve analysis based on threshold probability (49, 50). Decision curve analysis showed that if the threshold probability ranged from 20 to 80%, our nomogram will has a higher net benefit than the “treat-all,” “treat-none,” or CT-report scheme in LN status prediction.

For exosomal miRNA quantification, internal control for normalizing miRNA expression has been unclear (51). The most recognized internal control in cell/tissue RNU6B is undetectable in most exosomes even using RNA sequencing platform (52). In this study, we preliminarily selected miR-16 as the best endogenous miRNA control in exosome, which was stably expressed in exosome as described by *in vitro* experiment previously (35, 41). Furthermore, there were no statistical di?erences between the LN–, LN+, reflux esophagitis, Barrett's esophagus, esophageal leiomyoma, and the HD groups in the expression level of serum exosomal miR-16 in sequencing data and qRT-PCR data of our clinical serum exosomes, which supported the eligibility of miR-16 as an internal reference for the quantification of exosomal miRNAs. To date, the efficiency of miR-16 for exosomal miRNA normalization has indeed been examined in more than 400 samples and will be unceasingly evaluated in more enormous sample sizes and multiple medical centers.

MiRNAs are believed to be produced by multiple cell types and could regulate target gene expressions, as well as associated cellular progression (53). Previous studies demonstrated that dysregulation of miRNA expression could modulate tumor metastasis due to the communication in different types of tumor microenvironments cells by exosomes (54–56). In this study, bioinformation analysis was performed to elucidate the functions and mechanisms of the four exosomal miRNAs by GO and KEGG pathway analyses. Results showed the top 20 involved signaling pathways, including the cancer pathway, Ras signaling pathway, Rap1 signaling pathway, PI3K-Akt signaling pathway, and MAPK signaling pathway, suggesting that these four exosomal miRNAs play crucial roles in metastasis and proliferation progression of ESCC. Furthermore, in The Cancer Genome Atlas (TCGA) dataset, the expression levels of miR-432-5p in kidney renal papillary cell carcinoma (KIRP), kidney chromophobe (KICH), head and neck squamous cell carcinoma (HNSC), colon adenocarcinoma (COAD), and breast invasive carcinoma (BRCA) tumor tissues were significantly lower than those of adjacent normal tissues (all *P* < 0.05, Supplementary Figures 4A–E). However, there is no significant difference between esophageal carcinoma (ESCA) tumor tissues and adjacent normal tissues (*P* = 0.17, probably due to small sample size, Supplementary Figure 4F). Previous studies also showed that miR-432-5p negatively correlates with MGST3 expression in non-small cell lung cancer and inhibits drug efflux of cisplatin resistance by directly targeting MGST3 (57). Besides, miR-432-5p is reported to serve as a tumor suppressor in hepatocellular carcinoma and prostate cancer (58, 59) and could play important roles in regulating cell growth and metastasis of hepatocellular carcinoma cells (60). The results of these studies are consistent with our results that miR-432-5p plays tumor suppression role in cancer progression. These observations, combined with our findings, demonstrate that these four miRNAs could act as promising biomarkers for LN metastasis prediction and potential treatment target in ESCC patients. Although our clinical nomogram is promising, one limitation should be taken into consideration: serum samples of this study were only collected from Chinese patients, and the distribution of clinical characteristics might be not suitable for other races and regions. Thus, further multicenter prospective studies with intact follow-up information from diverse ethnic populations are required to validate whether our nomogram can be incorporated into routine clinical practice.

In conclusion, our results demonstrated that a novel serum exosomal miRNA-based nomogram was developed for the identification of LN metastasis. Our predictive nomogram also has excellent clinical value in non-invasive discrimination of patients with or without LN metastasis and may be conveniently used to improve overall patient treatment and outcomes in ESCC.

## Data Availability Statement

The datasets presented in this study can be found in online repositories. The names of the repository/repositories and accession number(s) can be found here: the NCBI Gene Expression Omnibus (GSE155360).

## Ethics Statement

The studies involving human participants were reviewed and approved by the Ethics Committee of The Second Hospital, Cheeloo College of Medicine, Shandong University. The patients/participants provided their written informed consent to participate in this study.

## Author Contributions

TL, L-TD, Y-SW, and C-XW conceived and designed the experiments. TL, Z-WS, and S-YG performed the experiments. TL, Y-SW, JL, and P-LL analyzed the data. TL, C-XW, JL, and HB wrote and revised the manuscript. TL, S-YG, and P-LL contributed to the sample collection and material support of this research. All authors provide final approval and agreed to be responsible for all aspects of the research. All authors contributed to the article and approved the submitted version.

## Conflict of Interest

The authors declare that the research was conducted in the absence of any commercial or financial relationships that could be construed as a potential conflict of interest.

## Supplementary Material

Supplementary Figure 1 Heatmap plot of 17 candidate miRNAs and the performance of four exosomal miRNAs in the validation cohort. **(A)** Heatmap plot of 17 candidate miRNAs. The relative expression level of exosomal chr 8-23234-3p **(B)**, chr 1-17695-5p **(C)**, chr 8-2743-5p **(D)**, and miR-432-5p **(E)** was examined using qRT-PCR. ROC curve analysis for the prediction of LN metastasis using chr 8-23234-3p **(F)**, chr 1-17695-5p **(G)**, chr 8-2743-5p **(H)** and miR-432-5p **(I)**. The relative expression level of exosomal chr 8-23234-3p **(J)**, chr 1-17695-5p **(K)**, chr 8-2743-5p **(L)** and miR-432-5p **(M)** in healthy donor (HD) and ESCC patients with (LN+) or without (LN-) LN metastasis were examined by qRT-PCR.

Supplementary Figure 2 Forest plot with odds ratio in training cohort. Univariate **(A)** and multivariate **(B)** logistic regression analysis for the LN metastasis associated four miRNAs in the training cohort. The bars represent the odds ratio (OR) and the 95% confidence interval (95% CI). **(C)** Gene ontology analysis of the four miRNAs.

Supplementary Figure 3 Top 20 significantly enriched pathway terms related to the four miRNAs.

Supplementary Figure 4 The expression level of miR-432-5p in TCGA dataset. **(A)** KIRP; **(B)** KICH; **(C)** HNSC; **(D)** COAD; **(E)** BRCA; **(F)**, ESCA.
