# Supplementary figures and images for: Development of a Novel Serum Exosomal MicroRNA Nomogram for the Preoperative Prediction of Lymph Node Metastasis in Esophageal Squamous Cell Carcinoma
Source: Front Oncol. 2020 Oct 6;10:573501. doi: 10.3389/fonc.2020.573501 (PMC7573187; doi:10.3389/fonc.2020.573501)

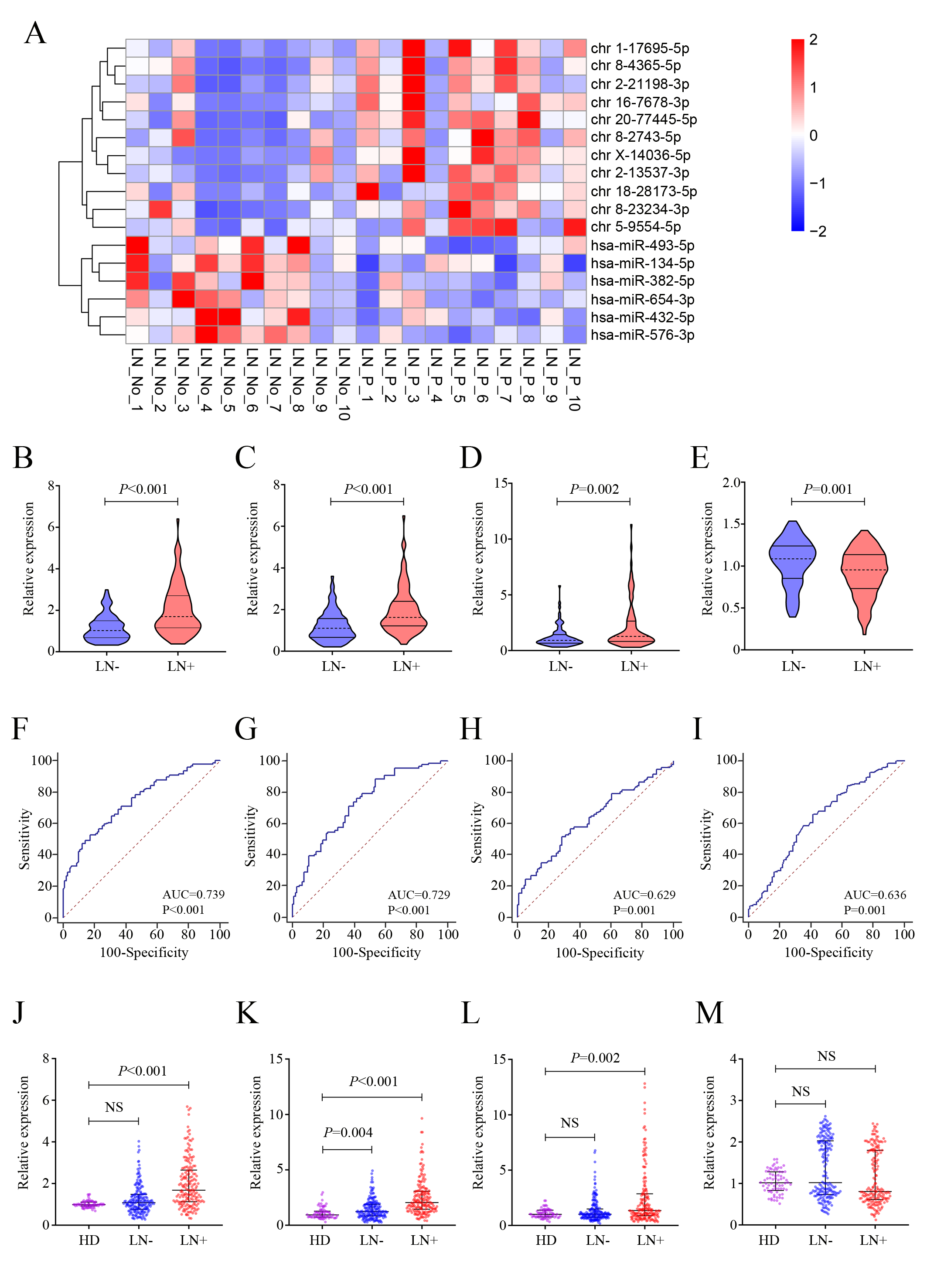

Supplement: Supplementary Figure 1 — Heatmap plot of 17 candidate miRNAs and the performance of four exosomal miRNAs in the validation cohort. (A) Heatmap plot of 17 candidate miRNAs. The relative expression level of exosomal chr 8-23234-3p (B), chr 1-17695-5p (C), chr 8-2743-5p (D), and miR-432-5p (E) was examined using qRT-PCR. ROC curve analysis for the prediction of LN metastasis using chr 8-23234-3p (F), chr 1-17695-5p (G), chr 8-2743-5p (H) and miR-432-5p (I). The relative expression level of exosomal chr 8-23234-3p (J), chr 1-17695-5p (K), chr 8-2743-5p (L) and miR-432-5p (M) in healthy donor (HD) and ESCC patients with (LN+) or without (LN-) LN metastasis were examined by qRT-PCR. [file Image_1.TIF]

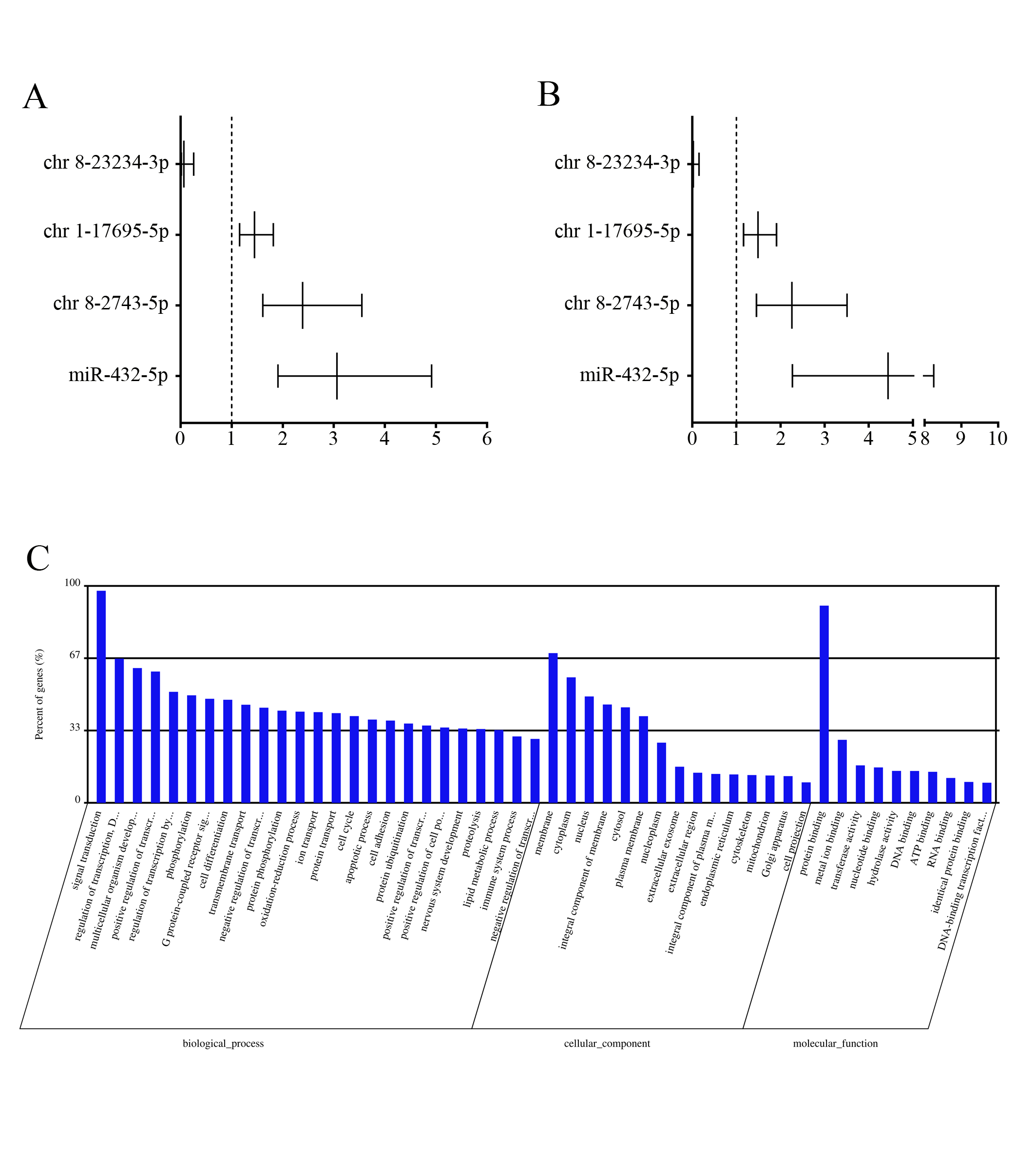

Supplement: Supplementary Figure 2 — Forest plot with odds ratio in training cohort. Univariate (A) and multivariate (B) logistic regression analysis for the LN metastasis associated four miRNAs in the training cohort. The bars represent the odds ratio (OR) and the 95% confidence interval (95% CI). (C) Gene ontology analysis of the four miRNAs. [file Image_2.TIF]

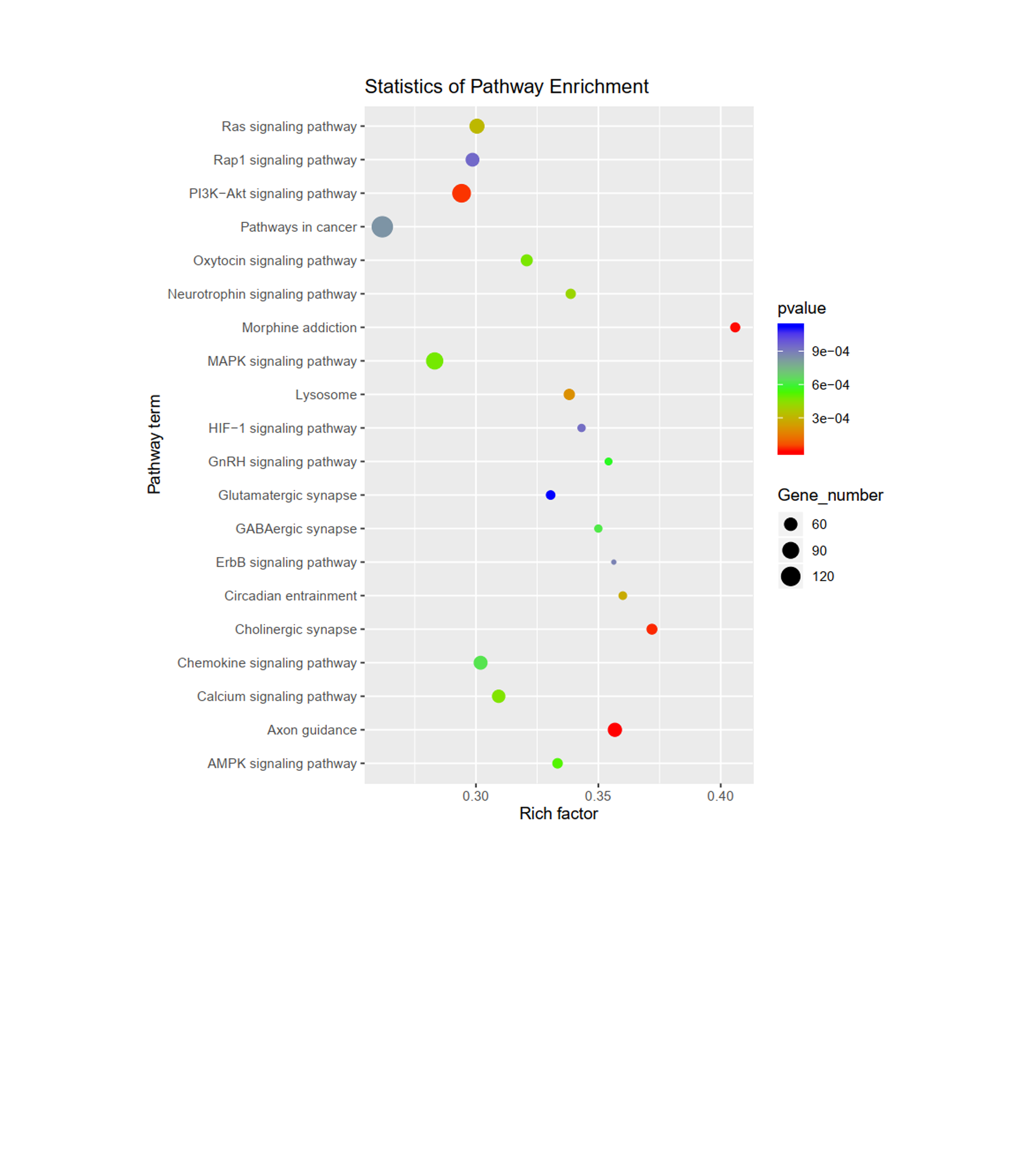

Supplement: Supplementary Figure 3 — Top 20 significantly enriched pathway terms related to the four miRNAs. [file Image_3.TIF]

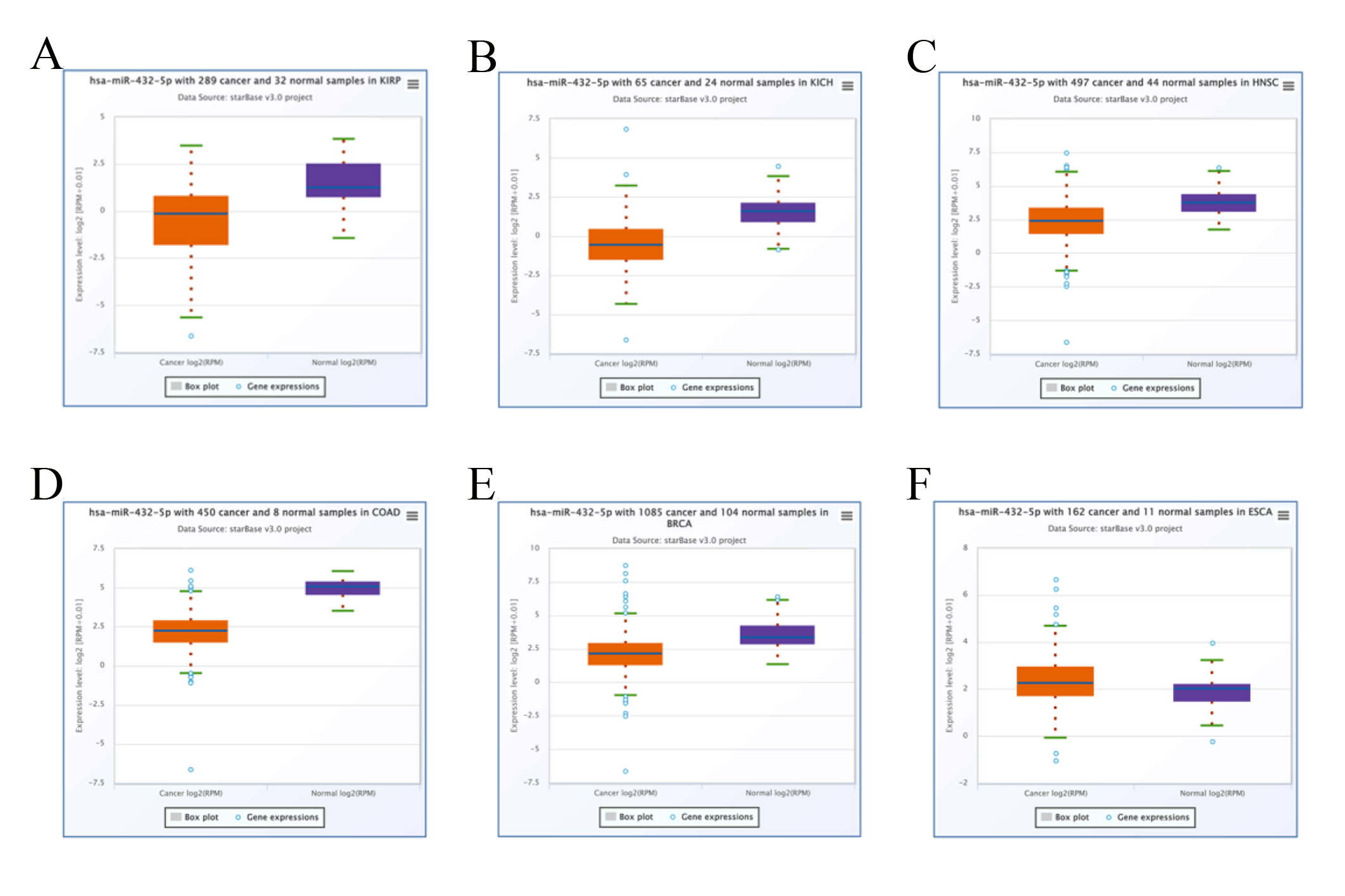

Supplement: Supplementary Figure 4 — The expression level of miR-432-5p in TCGA dataset. (A) KIRP; (B) KICH; (C) HNSC; (D) COAD; (E) BRCA; (F), ESCA. [file Image_4.TIF]
