# Supplementary material for: Development of a Novel Serum Exosomal MicroRNA Nomogram for the Preoperative Prediction of Lymph Node Metastasis in Esophageal Squamous Cell Carcinoma
Source: Front Oncol. 2020 Oct 6;10:573501. doi: 10.3389/fonc.2020.573501 (PMC7573187; doi:10.3389/fonc.2020.573501)
Supplement: Supplementary file 5 [file Table_1.docx]

**Table S1. Forward primer sequence of 17 candidate miRNA and internal control miR-16**

| **Index** | **miRNA name** | **Forward primer sequence** |
| --- | --- | --- |
| 1 | chr 20-77445-5p | GTGTTGGATGTCCCTGTTGCT |
| 2 | chr 1-17695-5p | GGAAACCGGATGCCTACAAAC |
| 3 | chr X-14036-5p | CTGATGGTGGGTTCCTTTGC |
| 4 | hsa-miR-134-5p | CGTGTGACTAGTTGACCAGAG |
| 5 | chr 16-7678-3p | GGGTGCTGACATGAGTAACG |
| 6 | chr 2-13537-3p | GTCTTCGTGGATGTCTAACC |
| 7 | hsa-miR-576-3p | GGCGATGTGGAAAAATTGGAAT |
| 8 | chr 2-21198-3p | TAGGTGGGAGACTATGAAACC |
| 9 | hsa-miR-493-5p | GGTACATGGTAGGCTTTCATT |
| 10 | chr 8-4365-5p | TGGATGAGCTGTGGATAGGG |
| 11 | chr 8-23234-3p | GCAGGTCCCAAGGGTATGAAA |
| 12 | chr 18-28173-5p | GAAACACAGCACTCTGCAAAC |
| 13 | chr 8-2743-5p | TTAGGTCTGGTGGAGTCGTC |
| 14 | hsa-miR-432-5p | TCTTGGAGTAGGTCATTGGGTGT |
| 15 | hsa-miR-654-3p | TATGTCTGCTGACCATCACC |
| 16 | hsa-miR-382-5p | AAGTTGTTCGTGGTGGATTCG |
| 17 | chr 5-9554-5p | GAGTACCTGCAGGAAAGGAC |
| 18 | miR-16 | CCTAGCAGCACGTAAATATTGG |
