# Supplementary material for: Development of a Novel Serum Exosomal MicroRNA Nomogram for the Preoperative Prediction of Lymph Node Metastasis in Esophageal Squamous Cell Carcinoma
Source: Front Oncol. 2020 Oct 6;10:573501. doi: 10.3389/fonc.2020.573501 (PMC7573187; doi:10.3389/fonc.2020.573501)
Supplement: Supplementary file 6 [file Table_2.doc]

**Table S2. Predictive performance for LN metastasis of individual biomarkers and miRNA-based panel in the training and validation cohorts.**

|  | **Cut-off value** | **Sensitivity%** | **Specificity%** | **AUC (95% CI)** |
| --- | --- | --- | --- | --- |
| **Training set** |  |  |  |  |
| chr 8-23234-3p | 1.035 | 77.1 | 61.1 | 0.725 (0.653 - 0.789) |
| chr 1-17695-5p | 1.240 | 71.1 | 66.3 | 0.726 (0.654 - 0.790) |
| chr 8-2743-5p | 1.042 | 60.2 | 66.3 | 0.621 (0.545 - 0.692) |
| miR-432-5p | 1.102 | 84.3 | 45.2 | 0.674 (0.600 - 0.742) |
| miRNA based panel | 0.437 | 79.5 | 82.1 | 0.865 (0.805 - 0.911) |
| **Validation set** |  |  |  |  |
| chr 8-23234-3p | 1.035 | 80.4 | 50.6 | 0.739 (0.670 - 0.800) |
| chr 1-17695-5p | 1.240 | 74.2 | 60.4 | 0.729 (0.659 - 0.791) |
| chr 8-2743-5p | 1.042 | 57.7 | 55.0 | 0.629 (0.556 - 0.698) |
| miR-432-5p | 1.102 | 58.8 | 64.8 | 0.636 (0.563 - 0.705) |
| miRNA based panel | 0.437 | 77.3 | 72.5 | 0.845 (0.785 - 0.893) |

The optimal cut-off values for each factor were determined using Youden’s index.
