# Supplementary material for: Development of a Novel Serum Exosomal MicroRNA Nomogram for the Preoperative Prediction of Lymph Node Metastasis in Esophageal Squamous Cell Carcinoma
Source: Front Oncol. 2020 Oct 6;10:573501. doi: 10.3389/fonc.2020.573501 (PMC7573187; doi:10.3389/fonc.2020.573501)
Supplement: Supplementary file 7 [file Table_3.docx]

| Clinical variable | Subgroups | Univariate analysis | | | Multivariate analysis | | |
| --- | --- | --- | --- | --- | --- | --- | --- |
|  |  | OR | 95% CI | P value | OR | 95% CI | P value |
| CT-report LN status | Positive vs Negative | 3.481 | 1.856 - 6.531 | <0.001 | 2.902 | 1.309 - 6.434 | 0.009 |
| SCC level | Abnormal vs Normal | 0.941 | 0.515 - 1.720 | 0.844 | 1.169 | 0.5220 to 2.6180 | 0.704 |
| CEA level | Abnormal vs Normal | 1.299 | 0.705 - 2.396 | 0.402 | 1.021 | 0.451 to 2.310 | 0.960 |
| miRNA-based Panel | High risk vs Low risk | 223.973 | 50.4737 - 993.861 | <0.001 | 191.016 | 42.2773 to 863.043 | <0.001 |

**Table S3. Univariate and multivariate logistic regression analysis of factors associated with lymph node metastasis in training cohort**

OR: odds ratio; CI: confidence interval.
